# Association between composite dietary antioxidant and bone mineral density in children and adolescents aged 8–19 years: findings from NHANES

**DOI:** 10.1038/s41598-024-66859-4

**Published:** 2024-07-09

**Authors:** Aiyong Cui, Juan Yan, Yuan Zeng, Baoqiang Shi, Long Cheng, Hongli Deng, Xing Wei, Yan Zhuang

**Affiliations:** 1 Department of Orthopaedics, Honghui Hospital, Xi’an Jiao Tong University, Xi’an, 710000 China; 2 Department of Medical Services Section, The Seventh Affiliated Hospital of Sun Yat-Sen University, Shen’zhen, 518107 China

**Keywords:** Composite dietary antioxidant index, Bone mineral density, Children, Adolescence, Endocrinology, Medical research

## Abstract

Dietary antioxidants may have beneficial effects on bone health, but it remains uncertain in children and adolescents. This study investigates the association of composite dietary antioxidant index (CDAI) with bone mineral density (BMD) in children and adolescents aged 8–19 years from the National Health and Nutrition Examination Survey (NHANES) 2007–2010. The study assessed the relationship between CDAI and BMD in 2994 individuals aged 8–19 years (average age 13.48 ± 3.32 years) from the NHANES 2007–2010. Multivariate linear regression analyses were utilized to detect the association between CDAI and total spine, femur neck, and total femur BMD, adjusting for confounders including age, race/ethnicity, sex, poverty income ratio (PIR), body mass index (BMI), serum phosphorus and calcium. Stratified analyses and interaction tests were performed to examine the stability of the results. The weighted characteristics showed that subjects in the fourth CDAI quartile were more likely to be older, men, and Non-Hispanic White. They have higher values of serum total calcium and phosphorus. After adjusting all confounders, CDAI was positively associated with the total spine (β = 0.0031 95% CI 0.0021–0.0040), total femur (β = 0.0039 95% CI 0.0028–0.0049), and femur neck BMD (β = 0.0031 95% CI 0.0021–0.0040) in children and adolescents. Furthermore, we found no interaction effects between different race/ethnicity, age, and sex groups. Our findings suggest that dietary intake of multiple antioxidants was positively associated with BMD in children and adolescents. These findings provide valuable evidence for improving bone health in the early stages of life. However, more prospective studies are required to validate our findings and their causal relationship.

## Introduction

Osteoporosis is a disease characterized by poor bone quality that leads to an increased risk of fractures and higher mortality in the elderly^1^. Using the diagnostic criteria of the World Health Organization, the global prevalence of osteoporosis in people aged > 50 was 20.5%^2^. Peak bone mass (PBM) accumulated in late adolescence is an important determinant of fragility fractures and osteoporosis risk in older adults^3^. Previous studies showed that a 10% increase in PBM during adolescence may decrease 50% fracture risk in senior citizens^4^. The formulation of PBM can be affected by nutrition, genetics, physical activities, certain diseases, and other factors^5–7^.

Reactive oxygen species (ROS) play a critical role in degenerative diseases like liver diseases, cardiovascular disease, osteoarthritis, and osteoporosis^8–10^. ROS accumulation is closely related to osteoclast and osteoblast apoptosis, impairing osteogenesis and mineralization of bone^11,12^. Moreover, redox imbalance caused by the excessive production of ROS increases macrophage osteoclast differentiation and supports an increase in bone loss, leading to osteoporosis^13^. Therefore, antioxidants have become a potential therapy to attenuate bone loss and prevent osteoporosis induced by excess ROS^11^. Several studies have explored the effects of ingesting individual antioxidants on bone mineral density (BMD), but the results are controversial^7,14–16^. A study by Kim et al.^17^ analyzed 1196 postmenopausal females from the Korean National Health and Nutrition Examination Survey (KNHANES) over 50 years. The findings suggested that dietary vitamin C intake was positively related to BMD. However, another study with 17 years of follow-up showed no protective effect of vitamin C supplementation on hip fracture risk^18^. These studies examining the impact of single antioxidant intake on BMD or osteoporosis were not representative of the total antioxidant intake and may produce biased results. The composite dietary antioxidant index (CDAI) is a standardized combination of intakes of six major antioxidants: vitamins C, E, and A, carotenoids, zinc, and selenium. It has been widely used as a composite score in studies involving the total dietary antioxidant capacity^19–21^. The CDAI has been proven to be closely associated with oxidative stress and inflammatory biomarkers (TNF-α and IL-1β)^22^. Recently, Liu et al.^23^ found a positive relationship between CDAI and BMD in U.S. adults. However, no studies have examined the association between CDAI and BMD in children and adolescents.

Adolescence is the most critical period for PMB formation and may largely influence the occurrence of osteoporosis or fragility fractures during adulthood or ageing. Nevertheless, the association between CDAI and BMD in children and adolescents is still unclear. This study is the first to explore the association between CDAI and BMD in individuals aged 8–19, aiming to provide crucial information on bone health in the early stages of life.

## Methods

### Study design

**Figure 1:**
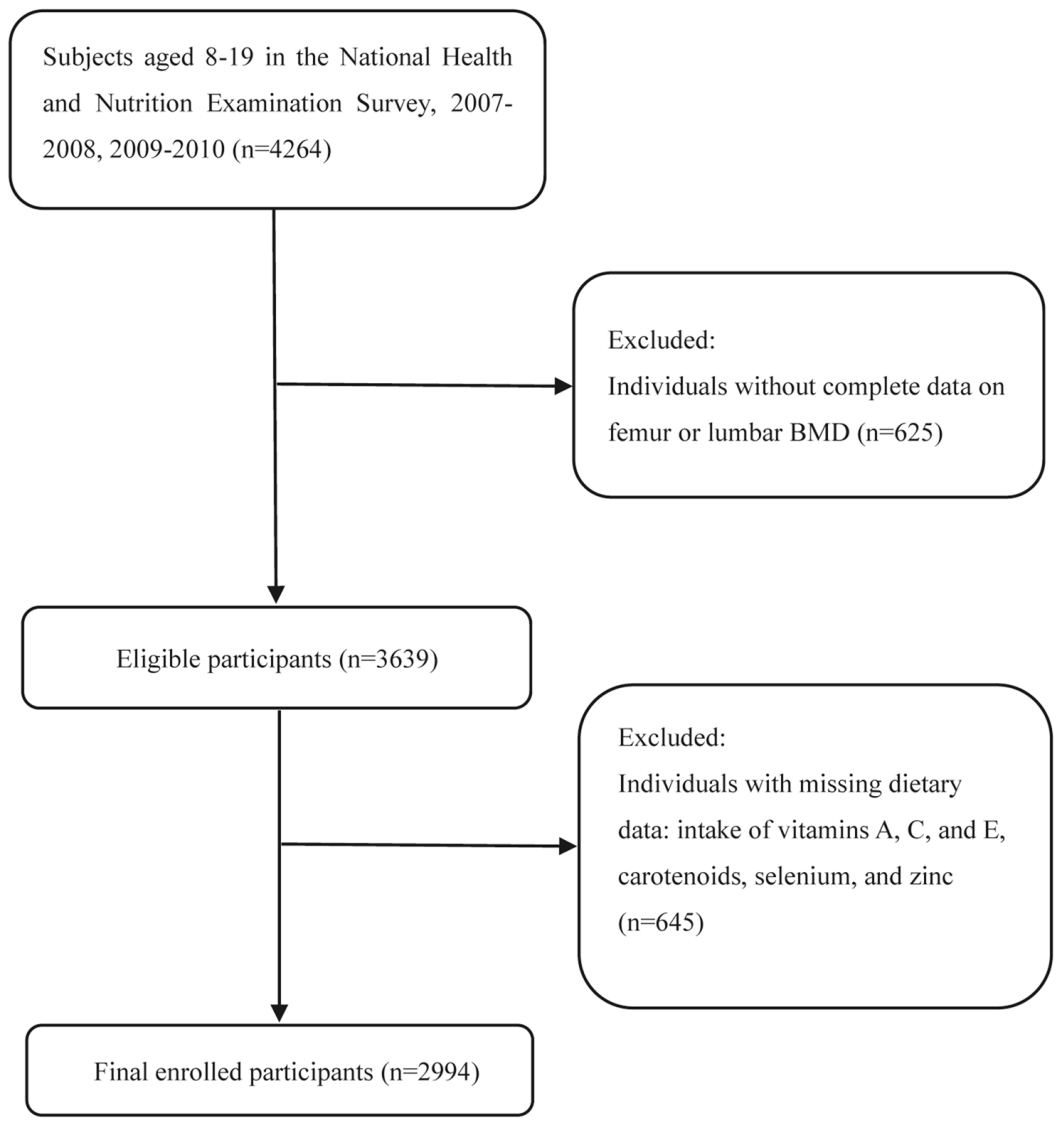
Flowchart of participants selection. *BMD* bone mineral density.

The NHANES is a national nutrition survey on the U.S. population that collects and publicly releases data biennially^24^. All participants or their guardians provided informed consent and the NHANES protocol was approved by the Research Ethics Review Board at NCHS. We combined data from the NHANES 2007–2008 and 2009–2010 because femoral BMD data of subjects less than 20 years only existed in these cycles. Then, children younger than 8 years were excluded since they did not receive the DXA examination in NHANES. Subjects with missing information on BMD (n = 625) and CDAI data (n = 645) were excluded. Finally, 2994 individuals aged 8–19 were enrolled in this study. The participant selection flowchart is displayed in Fig. 1.

### Variables

#### Composite dietary antioxidant index

Dietary intake for each subject in the NHANES dataset was documented through two 24-h dietary recall interviews with children and adolescents. The initial dietary recall interview was performed at the Mobile Examination Center (MEC), followed by a second interview 3 to 10 days later via a return phone call. The U.S. Department of Agriculture's Dietary Research Food and Nutrient Database was used to calculate antioxidant, micronutrient, and total energy intakes^25^. To calculate the CDAI, we summed up the intake of six antioxidants by averaging the two interviews (vitamins C, E, and A carotenoids, zinc, and selenium). These summed antioxidants were calculated from diet alone and did not include additional supplements/medications. Vitamin E and A intake were defined as equal milligrams of alpha-tocopherol and retinol. The six standardized dietary antioxidant intakes were calculated by deducting the mean and dividing by the standard deviation. Then, they were summed to obtain the CDAI scores^26^. The calculation formula is as follows: CDAI = ${\sum }_{i=1}^{n=6}x=\frac{\text{Individual \, Intake}-\text{ Mean}} \, {\text{SD}}$.

#### Bone mineral density

Dependent variables included total spine, femur neck, and total femur BMD, which were measured by dual-energy X-ray absorptiometry (DXA) using Hologic densitometers. Professionals collected and standardized the information on total spine, femur neck, and total femur BMD. Detailed BMD data can be accessed in DXXLSA, DXXOFBMD, and DXXNKBMD datasets on NHANES website^27^.

#### Covariates

Covariates were chosen based on the published studies to eliminate potential effects on the final results: race/ethnicity, age, sex, poverty income ratio (PIR), body mass index (BMI), serum phosphorus, and serum calcium^25,28,29^. Detailed data on BMD, CDAI, and additional covariates could be seen at the NHANES website (http://www.cdc.gov/nchs/nhanes/).

### Statistical analysis

All analyses were performed by R software (4.3.1) and EmpowerStats (4.1), using MEC weighted. A *P* value < 0.05 was regarded as significant. Percentages and mean ± standard deviation were used to represent categorical and continuous variables. To compare differences in continuous and categorical variables, weighted linear regression models and weighted χ^2^ tests were used, respectively. We employed weighted multivariate logistic regression analyses to evaluate the association of CDAI with total spine, femur neck, and total femur BMD. We first built an unadjusted model (Model 1). Then, Model 2 was created by adjusting race/ethnicity, age, and sex. Finally, Model 3 was created by adjusting the variables of race/ethnicity, age, sex, PIR, BMI serum phosphorus, and serum calcium. After dividing CDAI into quartiles, trend tests were utilized to analyze their linear association trend. Stratified analyses and interaction tests were performed by gender (male and female), age (8–13 and 14–19), and race/ethnicity (Mexican American, Non-Hispanic White, Other Hispanic, Non-Hispanic Black, and Other Race).

## Results

A total of 2994 subjects aged 8–19 years were recruited in this study, of which 52.39% were males and 47.61% were females with a mean age of 13.48 ± 3.32 years. In addition, 13.77% of the participants were Mexican American, 59.28% were non-Hispanic white, 13.94% were non-Hispanic black, 7.16% were other races (including multiracial), and 5.85% were other Hispanic. The weighted participants' characteristics were analyzed based on CDAI quartiles (Q1–Q4), as listed in Table 1. In the fourth quartile of CDAI group, participants were older, men, and race of Non-Hispanic White (*P* < 0.05). They have higher values of serum phosphorus and calcium.

**Table 1:** Weighted characteristics of the study population based on CDAI quartiles.

	Total	CDAI				*P* value
		Q1	Q2	Q3	Q4	
Number of subjects (n)	2994	749	748	748	749	
Age (years)	13.48 ± 3.32	13.37 ± 3.19	13.02 ± 3.40	13.24 ± 3.28	14.24 ± 3.26	< 0.001
Gender (%)						< 0.001
Men	52.39	35.86	46.61	52.59	71.98	
Women	47.61	64.14	53.39	47.41	28.02	
Race/ethnicity (%)						0.001
Mexican American	13.77%	15.82	13.81	11.52	12.45	
Other Hispanic	5.85%	5.92	6.57	6.38	6.20	
Non-Hispanic White	59.28%	53.31	59.24	61.37	64.51	
Non-Hispanic Black	13.94%	17.86	12.89	12.27	11.29	
Other race (including multi-racial)	7.16%	7.08	7.49	8.46	5.54	
BMI	22.12 ± 5.48	22.73 ± 5.94	21.61 ± 5.36	22.17 ± 5.64	22.03 ± 4.93	0.001
PIR	2.63 ± 1.63	2.30 ± 1.53	2.67 ± 1.67	2.81 ± 1.61	2.71 ± 1.65	
Serum total calcium (mmol/L)	9.62 ± 0.25	9.59 ± 0.24	9.63 ± 0.23	9.62 ± 0.26	9.64 ± 0.25	< 0.001
Serum phosphorus (mmol/L)	4.36 ± 0.54	4.36 ± 0.53	4.30 ± 0.51	4.41 ± 0.52	4.39 ± 0.57	0.001
Total spine BMD (g/cm^2^)	0.85 ± 0.20	0.85 ± 0.20	0.83 ± 0.20	0.85 ± 0.20	0.88 ± 0.19	< 0.001
Total femur BMD (g/cm^2^)	0.90 ± 0.19	0.88 ± 0.17	0.88 ± 0.19	0.90 ± 0.19	0.95 ± 0.19	< 0.001
Femur neck (g/cm^2^)	0.84 ± 0.17	0.82 ± 0.16	0.82 ± 0.17	0.84 ± 0.17	0.87 ± 0.17	< 0.001
Vitamin E (mg/day)	54.74 ± 27.92	30.18 ± 11.96	44.49 ± 13.47	57.74 ± 16.34	82.78 ± 31.01	< 0.001
Vitamin A (mcg/day)	601.03 ± 355.26	315.21 ± 175.04	489.01 ± 189.61	621.84 ± 236.20	934.29 ± 407.89	< 0.001
Vitamin C (mg/day)	76.68 ± 69.62	41.99 ± 36.99	62.18 ± 47.24	79.83 ± 62.20	117.39 ± 91.27	< 0.001
Carotenoid (mcg/day)	7513.86 ± 7775.57	3009.69 ± 2579.28	5244.15 ± 3917.62	7595.60 ± 5153.34	13,469.70 ± 11,124.26	< 0.001
Zinc (mg/day)	11.35 ± 6.39	6.57 ± 2.36	9.43 ± 2.60	11.77 ± 3.47	16.91 ± 8.72	< 0.001
Selenium (mcg/day)	103.40 ± 45.34	61.03 ± 18.75	86.56 ± 18.71	108.57 ± 24.98	151.04 ± 48.70	< 0.001

Mean ± SD for continuous variables: the *P* value was calculated by the weighted linear regression model. (%) for categorical variables: the *P* value was calculated by the weighted chi-square test. Abbreviation: *CDAI,* composite dietary antioxidant index. *BMD,* bone mineral density. *BMI*, body mass index. *PIR*, poverty income ratio.

### Association between CDAI and total spine BMD

**Figure 2:**
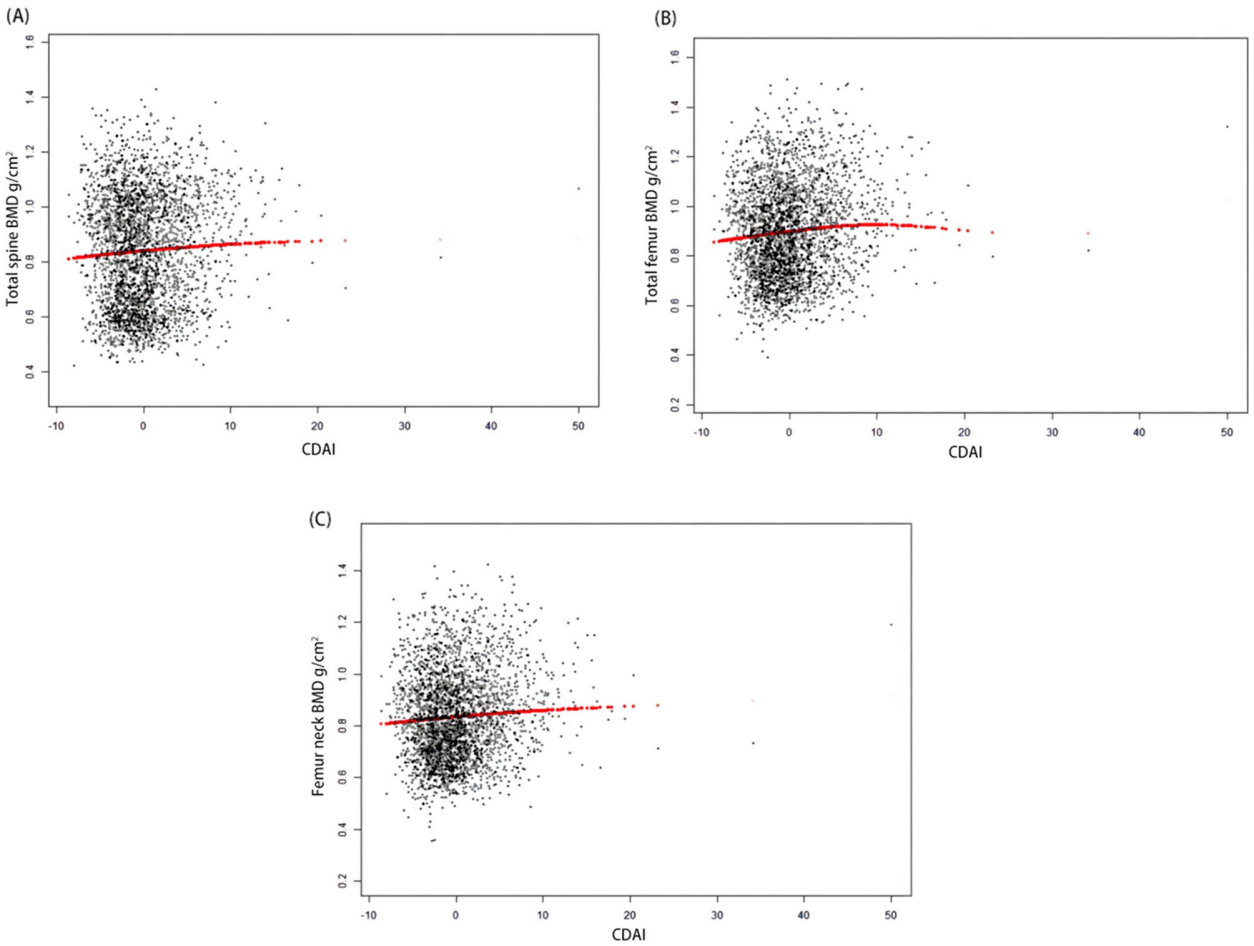
The association between CDAI and BMD. Each black point represents a sample. (**A**) CDAI on total spine BMD. (**B**) CDAI on total femur BMD. (**C**) CDAI on femur neck BMD. *CDAI* composite dietary antioxidant index, *BMD* bone mineral density.

**Figure 3:**
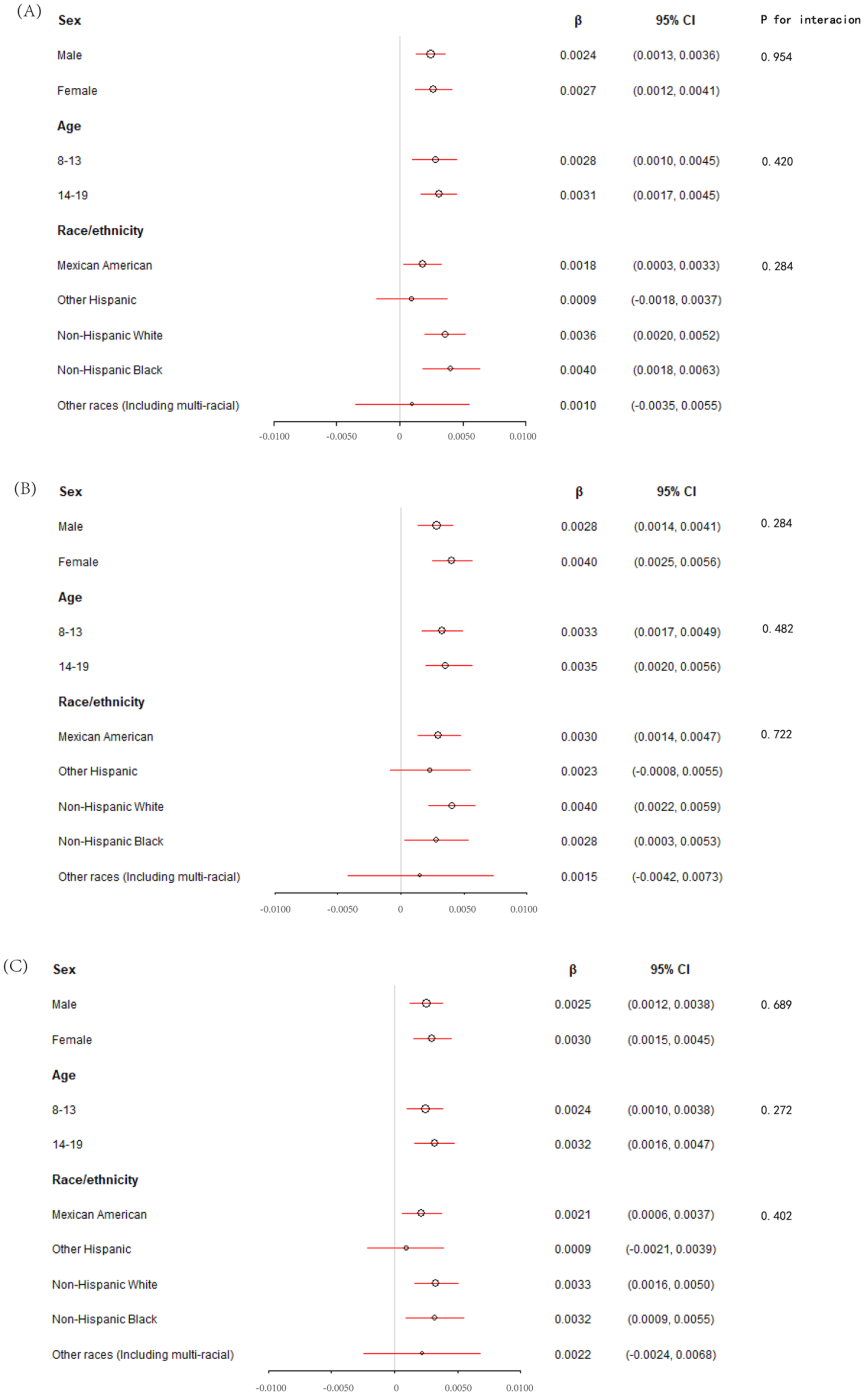
The results of stratified analyses. (**A**) CDAI on total spine BMD. (**B**) CDAI on total femur BMD. (**C**) CDAI on femur neck BMD. *CDAI* composite dietary antioxidant index, *BMD* bone mineral density.

The association between CDAI and total spine BMD can be found in Table 2 and Fig. 2A. The positive relationship between CDAI and total spine BMD was significant in both three models. In model 3, CDAI was positively associated with total spine BMD (β = 0.0031 95% CI 0.0021–0.0040). We divided the CDAI into quartiles and found that the lumbar BMD in subjects of the fourth CDAI quartile increases by 0.0325 g/cm^2^ than those in the first CDAI quartile (*P* for trend < 0.001). The results of stratified analyses are presented in Fig. 3A. After stratified by sex, we found no difference between males (β = 0.0024 95% CI 0.0013–0.0036) and females (β = 0.0027 95% CI 0.0012–0.0041) (*P* for interaction = 0.954). The positive association was significant in those aged 8–13 years (β = 0.0028 95% CI 0.0010–0.0045) and 14–19 years (β = 0.0031 95% CI 0.0017–0.0045) (*P* for interaction = 0.420). Then, we conduct a stratified analysis by race/ethnicity. Statistically significant correlation between CDAI and the lumbar BMD was found in non-Hispanic black (β = 0.0040 95% CI 0.0018–0.0063), non-Hispanic white (β = 0.0036 95% CI 0.0020–0.0052), and Mexican American (β = 0.0018 95% CI 0.0003–0.0033), but not in Other Hispanic (β = 0.0009 95% CI − 0.0018–0.0037) and other races (Including multi-racial) (β = 0.0010 95% CI − 0.0035–0.0055). However, the results showed no interaction effect between different race/ethnicity (*P* for interaction = 0.284).

**Table 2:** The association between CDAI and BMD in children and adolescents (g/cm^2^).

	Model 1 β (95% CI) *P* value	Model 2 β (95% CI) *P* value	Model 3 β (95% CI) *P* value
Total spine BMD			
CDAI	0.0039 (0.0021, 0.0057) < 0.001	0.0018 (0.0008, 0.0028) < 0.001	0.0031 (0.0021, 0.0040) < 0.001
CDAI categories			
Q1 (< 1.09)	Reference	Reference	Reference
Q2 (1.09–2.18)	0.0190 (− 0.0397, 0.0017) 0.071	0.0125 (0.0009, 0.0242) 0.035	0.0170 (0.0066, 0.0275) 0.001
Q3 (2.18–4.25)	− 0.0016 (− 0.0221, 0.0189) 0.878	0.0224 (0.0108, 0.0340) < 0.001	0.0257 (0.0152, 0.0361) < 0.001
Q4 (4.25–5.00)	0.0332 (0.0128, 0.0535) 0.001	0.0208 (0.0090, 0.0327) < 0.001	0.0325 (0.0218, 0.0431) < 0.001
P for trend	0.026	0.001	< 0.001
Total femur BMD			
CDAI	0.0074 (0.0057, 0.0090) < 0.001	0.0026 (0.0015, 0.0038) < 0.001	0.0039 (0.0028, 0.0049) < 0.001
CDAI categories			
Q1 (< 1.09)	Reference	Reference	Reference
Q2 (1.09–2.18)	0.0039 (− 0.0152, 0.0231) 0.686	0.0211 (0.0082, 0.0340) 0.001	0.0244 (0.0129, 0.0360) < 0.001
Q3 (2.18–4.25)	0.0263 (0.0073, 0.0453) 0.007	0.0319 (0.0190, 0.0448) < 0.001	0.0321 (0.0206, 0.0436) < 0.001
Q4 (4.25–5.00)	0.0754 (0.0566, 0.0942) < 0.001	0.0323 (0.0191, 0.0454) < 0.001	0.0434 (0.0317, 0.0552) < 0.001
P for trend	< 0.001	< 0.001	< 0.001
Femur neck BMD			
CDAI	0.0057 (0.0042, 0.0072) < 0.001	0.0017 (0.0006, 0.0028) 0.003	0.0031 (0.0021, 0.0040) < 0.001
CDAI categories			
Q1 (< 1.09)	Reference	Reference	Reference
Q2 (1.09–2.18)	− 0.0027 (− 0.0201, 0.0147) 0.761	0.0125 (− 0.0000, 0.0250) 0.050	0.0172 (0.0064, 0.0281) 0.002
Q3 (2.18–4.25)	0.0182 (0.0009, 0.0354) 0.039	0.0236 (0.0112, 0.0361) < 0.001	0.0246 (0.0138, 0.0354) < 0.001
Q4 (4.25–5.00)	0.0551 (0.0381, 0.0722) < 0.001	0.0190 (0.0063, 0.0318) 0.003	0.0316 (0.0206, 0.0427) < 0.001
*P* for trend	< 0.001	0.004	< 0.001

Model 1: no covariates were adjusted. Model 2: age and race/ethnicity were adjusted. Model 3: age, race/ethnicity, BMI, PIR, serum calcium, and serum phosphorus. *CDAI* composite dietary antioxidant index, *BMD* bone mineral density, *PIR* poverty income ratio, *BMI* body mass index.

### Association between CDAI and total femur BMD

The association between CDAI and total femur BMD can be found in Table 2 and Fig. 2B. The positive relationship between CDAI and total femur BMD was significant in both three models. In model 3, CDAI was positively associated with total femur BMD (β = 0.0039 95% CI 0.0028–0.0049). We divided the CDAI into quartiles and found that the total femur BMD in subjects of the fourth CDAI quartile increases by 0.0434 g/cm^2^ than in the first CDAI quartile (*P* for trend < 0.001). The results of stratified analyses are presented in Fig. 3B. After stratified by sex, we found no difference between males (β = 0.0028 95% CI 0.0014–0.0041) and females (β = 0.0040 95% CI 0.0025–0.0056) (*P* for interaction = 0.284). The positive association was significant in those aged 8–13 years (β = 0.0033 95% CI 0.0017–0.0049) and 14–19 years (β = 0.0035 95% CI 0.0020–0.0056) (*P* for interaction = 0.482). Statistically significant correlation between CDAI and the total femur BMD was found in non-Hispanic black (β = 0.0028 95% CI 0.0003–0.0053), non-Hispanic white (β = 0.0040 95% CI 0.0022–0.0059), and Mexican American (β = 0.0030 95% CI 0.0014–0.0047), but not in Other Hispanic (β = 0.0023 95% CI − 0.0008–0.0055) and other races (Including multi-racial) (β = 0.0015 95% CI − 0.0042–0.0073). However, the results showed no interaction effect between different race/ethnicity (*P* for interaction = 0.722).

### Association between CDAI and femur neck BMD

The association between CDAI and femur neck BMD can be found in Table 2 and Fig. 2C. In both three models, the positive relationship between CDAI and femur neck was significant. In model 3, CDAI was positively associated with femur neck BMD (β = 0.0031 95% CI 0.0021–0.0040). We divided the CDAI into quartiles and found that the total femur BMD in subjects of the fourth CDAI quartile increases by 0.0316 g/cm^2^ than in the first CDAI quartile (*P* for trend < 0.001). The results of stratified analyses are presented in Fig. 3C. After stratified by sex, we found no difference between males (β = 0.0025 95% CI 0.0012–0.0038) and females (β = 0.0030 95% CI 0.0015–0.0045, *P* < 0.001) (*P* for interaction = 0.689). The positive association was significant in those aged 8–13 years (β = 0.0024 95% CI 0.0010–0.0038) and 14–19 years (β = 0.0032 95% CI 0.0016–0.0047) (*P* for interaction = 0.272). Statistically significant correlation between CDAI and the femur neck BMD was found in non-Hispanic black (β = 0.0032 95% CI 0.0009–0.0055), non-Hispanic white (β = 0.0033 95% CI 0.0016–0.0050), and Mexican American (β = 0.0021 95% CI 0.0006–0.0037), but not in Other Hispanic (β = 0.0009 95% CI − 0.0021–0.0039) and other races (Including multi-racial) (β = 0.0022 95% CI − 0.0024–0.0068). However, the results showed no interaction effect between different race/ethnicity (*P* for interaction = 0.402).

## Discussion

Our study is the first to explore and identify a positive association between CDAI and BMD in children and adolescents. This positive association remained stable across age, gender, and race/ethnicity. The results of this study provide evidence supporting the role of antioxidant intake in the early prevention of osteoporosis.

In recent years, dietary intake of antioxidants has been shown to be associated with a variety of health factors, including bone health^30–32^. In an early study in the United States, Morton et al.^33^ found that vitamin C supplements were beneficial for increasing BMD of the femur, femoral neck, and radius in postmenopausal women. In another four-year study, Sahni et al.^34^ found that carotenoid intake could protect bone health in an elderly population. Wu et al.^35^ found that a higher selenium status is independently related to an osteoporosis risk in subjects aged > 40 years. However, there are few studies on the effects of antioxidant intake on bone health in children and adolescents. Furthermore, these studies always focus on a single antioxidant and ignore the overall total dietary antioxidant capacity^7,36–38^. For example, in a cross-sectional study of 426 children, Zhang et al.^36^ found that vitamin A intake is positively related to BMD after adjusting confounders. However, in another study involving 888 subjects aged 15–19 years, Teigmo et al.^39^ found no association between vitamin A status and BMD. Selenium is believed to play a role in bone health because of its antioxidant ability. In a recent NHANES study, the dose–response analyses showed an inversed U-shaped association between selenium status and BMD in individuals aged 8–19 years. Appropriate selenium intake benefits children's bone health, while excessive selenium may exert adverse effects on children's bone health^37^. These studies involving a single antioxidant may ignore overall total dietary antioxidant capacity as some dietary antioxidants require interactions for synergistic effects^40^. A study by Turan et al.^41^ found that the concomitant use of selenium with vitamins E and C prevented osteoporosis in a rabbit model. The combination effect of these antioxidants was better than that of the single one^41^. The latest review indicated that separate antioxidants may help bone health, while multiple antioxidants from whole plant foods may have more overall benefits^42^. The CDAI considers dietary intake of various antioxidants such as minerals (zinc, selenium), vitamins (vitamin C, vitamin E), and phytochemicals (flavonoids, carotenoids) and is able to adequately respond to the antioxidant capacity of our diet. In a previous study, Liu et al.^43^ found a positive relationship between CDAI and femur BMD in American adults aged ≥ 20 years. Their results showed that every unit increase in dietary CDAI was associated with 0.003 and 0.001 g/cm^2^ increase in femoral neck and total spine BMD, respectively. Similar to adults, our study also confirmed the positive association between CDAI and BMD in children and adolescents. Our study showed that every unit increase in dietary CDAI was associated with 0.0039 and 0.0031 g/cm^2^ increase in femur neck and total spine BMD, which is larger than in adults. Thus, combined intake of dietary antioxidants may contribute to bone health in adolescents.

Although there is no direct evidence on the mechanism of CDAI on bone health in children and adolescents, several cellular or animal studies have explained the positive effects of antioxidants on bone health^30,44^. Oxidative stress is an imbalance of oxidative and antioxidant effects that tends to oxidize in our body and is an important contributor to ageing and disease, including osteoporosis^45^. Excess ROS generated by oxidative stress imbalance could inhibit the expression of osterix and Runx2, thereby reducing osteogenic activity^46^. The evidence suggested that osteoclastogenic markers like TRAP, NFATc1, and c-Fos were upgraded after ROS induction^47^. In addition, the researcher also found decreased cell viability, increased lipogenic differentiation, and increased osteogenic differentiation in H2O2-treated bone marrow mesenchymal stem cells (BM-MSCs)^48^. Moreover, NADPH oxidase 4 (NOX4) is an essential source of active enzymes that make up ROS. Goettsch et al.^49^ found that NOX4 was overexpressed in patients with increased osteoclast activity. Furthermore, their results suggested a reduced bone loss in Nox4 knockdown or pharmacologically suppressed ovariectomized mice.

In total, we first evaluated and found a positive association of dietary antioxidant exposure with bone health in children and adolescents. To ensure the stability of the findings, we performed subgroup analyses. The results showed that the positive association between CDAI and BMD remained stable across gender and age. Interestingly, though results showed no interaction effect between different race/ethnicity, we found that the positive correlation was significant only among non-Hispanic whites, Mexican Americans, and non-Hispanic blacks. Genetic differences between races may partly account for these differences, as studies suggest that 50% to 85% of PBM is genetically determined^50^. However, more investigations are required to validate our findings.

Our results have several advantages. First, this is a large sample analysis from NHANES survey. All analyses used MEC sampling weights that are representative of the general population in the United States. Second, we found that CDAI was positively associated with lumbar and femoral BMD. The use of CDAI as an exposure variable rather than a single antioxidant provides valuable evidence for the association between dietary antioxidants and bone health during adolescence. Third, we used dietary data that were the average of two 24-h dietary measurements, thereby increasing the reliability of the results. Our study also has some limitations. First, it is hard to infer causality because of the nature of the cross-sectional study. Second, our study population did not include individuals aged less than 8 years since their BMD data were not available in NHANES. Third, we tried to adjust for some of the confounders. This may only partially rule out the effect of confounders on the final results. Covariates such as children's physical activity and vitamin D status were not available in these NHANES cycles and thus may have affected the stability of the results. Fourth, CDAI may not be representative of the total intake of antioxidant ability. Future research may require a more representative indicator of the overall antioxidant activity of these compounds.

## Conclusions

Our findings suggest that dietary intake of multiple antioxidants was positively associated with BMD in children and adolescents. These findings provide valuable evidence for improving bone health in the early stages of life. However, prospective studies are required to validate our findings and their causal relationship.

## Data Availability

The datasets generated during and/or analysed during the current study are available in the [NHANES] repository, [https://www.cdc.gov/nchs/nhanes/].
